# Remarkable Results of the New German Cæsarean Operations

**Published:** 1887-08

**Authors:** 


### REMARKABLE RESULTS OF THE NEW GERMAN CÆSAREAN OPERATIONS.

These distinct methods of performing or completing the Cæsarean section have been devised and tested in Germany during the last five years; two of them by their respective originators, and the third likewise, and also by several operators, who have been influenced by its designer to adopt his method. The cases in which this operation has been performed, amounting to seventeen in number, have had a far less fatality than has followed in the same country, either the old Cæsarean section or its modification by utero-ovarian amputation.

The processes which bear the names, respectively, of Frank, Kehrer and Sanger, have been fully described in *The American Journal of the Medical Sciences* for July, 1883, page 232, to-wit:

*Frank's Method.*—Wash the abdomen with ether and with a 5 per cent. solution of carbolic acid; disinfect the vagina by irrigating with the latter fluid. Turn out the uterus entire, and incise it vertically, commencing low down in the vesico-uterine excavation; extract the fœtus and secundines; wash the front of the uterus, its interior and the vagina with a 5 per cent. carbolic acid solution. Pass a large drainage tube through the abdominal and uterine wounds and out through the vagina. Suture the uterus above the tube with strong catgut. Draw the round ligaments together above the uterine wound, and secure them with sutures of Czerny silk, so as to close over and separate from the abdominal cavity the vesico-uterine pouch, which is to be drained by three tubes—"one utero-vaginal, one pre-uterine, and a third applied along the uterine wound to the top of the pavilion."

*Process of Kehrer.*—Open the abdomen through the linea alba. Incise the uterus transversely between the insertions of the round ligaments. Extract the fœtus and secundines; close the muscular layer of the uterus by from six to ten deep-seated stitches of carbolized silk, and the peritoneal portion by from twelve to twenty-five. Use Listerism in the operation and dressings, abdominal drainage and vaginal irrigation.

*Process of Sanger.*—Abdominal incision to be made as usual; two strong ligatures are to be inserted through the margins of the wound near its upper angle to be drawn upon after turning out the uterus. Membranes to be ruptured through the vagina. If practicable, the uterus is to be lifted out and held vertically. A sheet ot caoutchouc, moistened with a 5 per cent. solution of carbolic acid, is to be made to inclose the cervix and cover the abdomen; to protect its cavity against the entrance of fluid. The ligatures are to be drawn upon to close the abdominal wound, while the uterus is incised *in sutu*, manual compression is to be made as a hæmostatic upon its lower segment; if opened after being turned out, manual compression: the application of clamps to the broad ligaments, or of an elastic tube to the cervico-uterine cone. After evacuating the uterus, any hæmorrhage from the wound is to be checked by hæmostatic pincettes. When the uterus has well contracted, pass a utero-vaginal drainage-tube, and introduce a carbolized sponge into the uterine cavity. Dissect the peritoneum free from the muscular edges of the uterine wound, and pare from the latter on each side a long slice of tissue of a wedge-shape, the thick edge being next to the peritoneal side, and the thin edge to the uterine cavity. The free edges of the peritoneum are now to be turned in over the muscular layer, and deep-seated stitches of silver wire or silk inserted, so as to penetrate the peritoneum and pass nearly through the muscular coat. Then superficial stitches at short intervals are to be passed, so as to secure the turned in peritoneum and keep its serous surfaces in contact, making a secure welt.

Frank has operated, twice, viz: on August 9, 1881, and December 8, 1884, losing the first patient, who died of shock in ten hours, and saving the second. Kehrer has had four cases, viz: on September 25, 1881; November 13, 1881; April 11, 1882, and June 20, 1885. The first and fourth recovered; the second died of septic peritonitis in fifty-three hours, and the third of septicæmia in five days. The Sanger operation has been performed in Germany alone eleven times, five times by Leopold, of Dresden, the first on May 25, 1882, with the loss of one case, his fourth, operated upon November 28, 1884, which died in five days of septic peritonitis. Sanger has operated four times in Leipzig, the first on November 16, 1884, and the other three last year. These patients all recovered. A tenth Sanger operation was that of Beumer, which was unfortunately fatal in forty hours, the woman being found upon autopsy to have had a pyelonephritis; this operation was in Griefswald. The eleventh case was under the care of Oberg, of Hamburg, and was operated upon on July 17, 1884; the patient recovered.

We have, then, seventeen of the new German Cæsarean operations to be placed to the credit of Germany, with the saving of thirteen women, or 76 6-17 per cent. By the Sanger operation alone, we have the remarkable result of 81 9-11 per cent. of women saved, and 100 per cent. of the children, which is considerably more than double the saving effected by the Porro method in Germany. To find an equivalent of nine women saved by the Cæsarean section in the United States, we must go back over a period of eleven years, and examine the records of thirty-nine operations, by which thirty women and twenty children were lost. This frightful mortality, of 76 12-13 per cent. of women is far greater than was encountered thirty years ago in our country, and is largely due to the fact that the Cæsarean operation is believed to be almost inevitably mortal, and hence is not resorted to until the patient is in an almost hopeless state. We have made the operation by delay as fatal as our English text-books teach us it is in Great Britain; and have done this, too, notwithstanding the fact that early operations have saved 75 per cent. of our women.—*Medical News*.

